# *Rosa rugosa* Root Extract as a Functional Food Ingredient, Mitigating UVB‐Induced Skin Aging Through MAPK/AP‐1 and AGE‐RAGE Pathway Modulation

**DOI:** 10.1002/fsn3.72462

**Published:** 2026-10-28

**Authors:** Min Ji Gu, Guijae Yoo, Sang‐Hoon Lee, Ho‐Young Park, Yoonsook Kim, Donghwan Kim, Sang Keun Ha

**Affiliations:** ^1^ Food Functionality Research Division Korea Food Research Institute Wanju‐gun Jeonbuk‐do Republic of Korea; ^2^ Division of Food Biotechnology University of Science and Technology Daejeon Republic of Korea

**Keywords:** advanced glycation end‐products, inflammation, *Rosa rugosa* root, skin photoaging, ultraviolet

## Abstract

*Rosa rugosa* root extract (RR‐r) has been used in East Asian medicine to treat metabolic disorders, including diabetes and alcohol‐induced liver toxicity. Although RR‐r has been demonstrated to exhibit anti‐inflammatory and antioxidant properties, further research is required to elucidate its protective effects against ultraviolet B (UVB)‐induced skin photoaging. In this study, high‐performance liquid chromatography was used to identify rutin as the major indicator compound of RR‐r. The photoprotective activity of RR‐r was evaluated using a mouse model of UVB‐induced skin damage. Mice were orally administered RR‐r (100 or 200 mg/kg) for 8 weeks during UVB irradiation. RR‐r significantly alleviated UVB‐induced wrinkle formation, epidermal thickening, and collagen fiber degradation. The assessment of skin morphology and hydration was conducted through the use of histological staining (H&E and Masson's trichrome), wrinkle and moisture analysis, and enzyme‐linked immunosorbent assay quantification of hyaluronic acid. Western blotting revealed that RR‐r increased pro‐collagen type I, tissue inhibitor of metalloproteinases 1, hyaluronan, collagen‐1 while reducing the matrix metalloproteinase family, hyaluronidase 1 and hyaluronidase 2. A further mechanistic analysis revealed that RR‐r inhibited UVB‐induced phosphorylation of mitogen‐activated protein kinase pathway components and downstream activator protein‐1 factors. Furthermore, RR‐r significantly suppressed UVB‐induced accumulation of advanced glycation end products (AGEs) and expression of their receptor, RAGE. Collectively, these findings demonstrate that oral RR‐r effectively protects against UVB‐induced photoaging by regulating wrinkle‐related proteins, enhancing skin hydration, and modulating MAPK/AP‐1 and AGE‐RAGE signaling. Thus, RR‐r may represent a promising natural candidate for skin anti‐aging interventions.

## Graphical abstract

*Rosa rugosa* root extract (RR‐r) attenuated UVB‐induced wrinkle formation and collagen degradation while improving skin hydration. Its photoprotective effects were associated with modulation of MAPK/AP‐1 and AGE‐RAGE signaling.

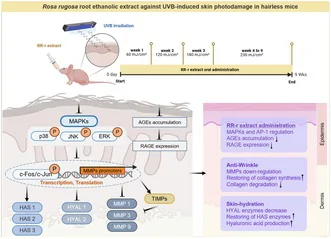

## 1 Introduction

Increasing life expectancy and the growing elderly population have heightened interest in age‐related physiological changes and diseases that affect quality of life, including skin health (Machado et al. 2023). The skin plays a critical protective role by shielding internal organs from external stressors such as ultraviolet (UV) radiation, smoking, and environmental pollutants, while also being influenced by intrinsic factors such as genetic aging processes (Flores‐Balderas et al. 2023; McCullough and Kelly 2006). Consequently, interest in strategies to maintain skin health and appearance has increased, driving demand for anti‐aging and photoprotective cosmetics, functional foods, and dietary supplements derived from natural materials (Cao et al. 2020; Cavinato et al. 2017). In this context, plant‐based diets and bioactive phytochemicals have been actively investigated for their protective effects against inflammatory and oxidative skin damage (Ittycheri et al. 2023).

Among extrinsic factors, UV radiation is a major contributor to skin photoaging. Although moderate UV exposure contributes to vitamin D synthesis and may benefit mental health (Humble 2010), excessive and chronic UV exposure induces oxidative stress by generating reactive oxygen species (ROS) (X. Chen et al. 2021). Increased ROS activates intracellular signaling pathways, including mitogen‐activated protein kinases (MAPKs) and activator protein‐1 (AP‐1), leading to the upregulation of matrix metalloproteinases (MMPs) (Pittayapruek et al. 2016). Elevated MMP expression promotes the degradation of extracellular matrix (ECM) components such as collagen, elastin, fibronectin, and proteoglycans, resulting in wrinkle formation and loss of skin structural integrity (Quan et al. 2009). In particular, MMPs have been identified as wrinkle regulators in skin aging. Chronic UV irradiation has been shown to induce the degradation of collagen, a key factor in the aging of the skin, and photodamage caused by MMPs (B. Chen et al. 2025). Ultraviolet‐B (UVB) exposure disrupts skin hydration by altering hyaluronic acid (HA) metabolism through dysregulation of hyaluronan synthases (HASs) and hyaluronidases (HYALs) (Song et al. 2022).

Collectively, UV‐induced ECM degradation, reduced skin hydration, and increased inflammatory responses accelerate skin aging. These processes are mediated by key molecular regulators, such as MMPs, HASs, and HYALs, which contribute to the structural breakdown and functional impairment of the skin barrier. More recently, accumulating evidence has suggested that advanced glycation end‐products (AGEs) and the activation of their receptor (RAGE) further exacerbate UV‐induced skin damage. The AGE‐RAGE signaling axis promotes chronic inflammation and oxidative stress, forming a self‐amplifying cycle that accelerates photoaging (Gu et al. 2023). The AGE‐RAGE axis enhances oxidative stress and chronic inflammation, forming a positive feedback loop that accelerates skin photoaging (Sultana et al. 2024). Therefore, targeting the AGE‐RAGE signaling pathway may represent a promising therapeutic strategy for preventing UV‐induced skin aging.

*Rosa rugosa*, a member of the family Rosaceae, is widely distributed in East Asia and has traditionally been used in herbal medicine for the treatment of various conditions, including diabetes and alcohol‐induced liver toxicity (Zhang et al. 2024). Previous studies have reported that *R. rugosa* root extract (RR‐r) exhibits antioxidant activity and inhibits lipid accumulation (D. H. Choi et al. 2022), and that its ethanol extract attenuates amyloid beta‐induced neurotoxicity and improves behavioral outcomes (C. K. Park et al. 2025). In addition, preliminary findings suggested that RR‐r modulates oxidative stress‐ and aging‐related pathways (L. Liu et al. 2018). Despite these reported biological activities, the potential protective effects of RR‐r against UVB‐induced skin photoaging have not yet been systematically investigated. In particular, no previous study has examined whether RR‐r modulates the AGE‐RAGE signaling axis in the context of UVB‐induced skin damage. Furthermore, in vivo evidence demonstrating its effects on skin morphology and photoaging‐related molecular markers remains limited. Therefore, the present study aimed to investigate whether RR‐r protects against UVB‐induced skin photoaging in vivo using an HR‐1 hairless mouse model, and to clarify its underlying molecular mechanisms. In particular, this study focused on collagen metabolism, skin hydration, and wrinkle formation, while specifically examining the involvement of MAPK/AP‐1 and the AGE‐RAGE signaling axis, which has not previously been explored in the context of RR‐r‐mediated photoprotection.

## 2 Materials and Methods

### 2.1 Preparation of RR‐r and High‐Performance Liquid Chromatography (HPLC) Analysis

RR‐r was obtained from a commercial market (Gyeongdong, Seoul, Republic of Korea). The RR‐r sample was identified by Dr. Guijae Yoo, and a voucher specimen (KF0122) was deposited at the Korea Food Research Institute. To extract the RR‐r, roots were dried, crushed to powder, and then extracted with 10 volumes of 70% ethanol (1:10, w/v) at 50°C for 3 h. The extraction process was repeated twice. After extraction, RR‐r extracts were filtered and then concentrated using rotary evaporation under reduced pressure (R‐114 Rotavapor system, Büchi Labortechnik AG, Flawil, Switzerland). Subsequently, the concentrate was lyophilized and stored at −20°C until use in experiments. The RR‐r ethanolic extract was performed with the utilization of an HPLC. The HPLC was recorded under the following conditions: model–Waters e2695 HPLC system; column: YMC Hydrosphere C18 (150 × 4.6 mm, 5 μm); detector: UV–Vis; mobile phase: (A) 0.1% formic acid in water with a gradient elution of 80%–10%, and (B) 0.1% formic acid in acetonitrile with a gradient elution of 20%–90%; injection volume: 10 μL; method time: 30 min; and flow rate: 0.4 mL/min; detector: UV (at 250 nm).

### 2.2 Animal Experiments

Male hairless mice (6 weeks old) were obtained from Central Lab. Animal Inc. (Seoul, Korea). The mice were housed at 23°C ± 1°C, with a humidity of 50% ± 5%, and a 12 h light/dark cycle. After a 1 week acclimation period, the mice were randomly divided into five groups (*n* = 8 per group): (i) normal control group (UVB non‐irradiated control; NC), (ii) UVB‐irradiated control group (Con), (iii) UVB‐irradiated group administered RR‐r 100 mg/kg/day (R100), (iv) UVB‐irradiated group administered RR‐r at 200 mg/kg/day (R200), and (v) UVB‐irradiated positive control group (Phosphatidylserine, 50 mg/kg/day). Distilled water was used as the vehicle, and the samples were administered orally for 9 weeks. The mice were randomly assigned to groups using a randomized allocation schedule. The investigators responsible for outcome assessment and data analysis were blinded to group allocation. All animal experiments were conducted in accordance with the guidelines of the Institutional Animal Care and Use Committee and approved under animal protocol (approval no. IV‐RA‐16‐2006‐22). The present study was conducted as an independent experiment within the scope of the approved protocol and in accordance with the Laboratory Animals Welfare Act of Korea and the Korean national laws for animal welfare. Furthermore, in accordance with the ARRIVE guidelines 2.0 and the Guide for the Care and Use of Laboratory Animals, all experimental procedures were performed to ensure the ethical treatment and welfare of the animals. During the experimental period, the animals were provided with food and water *ad libitum*, and were monitored regularly for their general health status.

### 2.3 UVB‐Induced Photoaging Model

To induce chronic skin photoaging, the UVB irradiation protocol was based on previously established UVB‐induced photoaging protocols in hairless mice, with minor modifications (Choi et al. 2019; Jung et al. 2023; Kim et al. 2016). Briefly, the dorsal skin of HR‐1 hairless mice was irradiated using a UVB device (UVP CL‐1000M UV Crosslinker; UVP Inc., CA, USA) three times per week for 9 weeks. The UVB dose was progressively increased from 60 mJ/cm^2^ during week 1–120 mJ/cm^2^ during week 2, and 180 mJ/cm^2^ during week 3, followed by 230 mJ/cm^2^ from week 4–9 at 302 nm. The cumulative UVB dose over the 9‐week experimental period was 5220 mJ/cm^2^. This stepwise UVB exposure was used to establish chronic photoaging while limiting excessive acute photodamage and has been shown to induce characteristic photoaging changes, including wrinkle formation, epidermal thickening, and collagen degradation.

### 2.4 Animal Grouping and Dosing

The doses of RR‐r (100 and 200 mg/kg/day) were selected based on dose ranges used for orally administered natural extracts reported in previous animal studies (Kim et al. 2024; Kim et al. 2023; Zhu et al. 2026). To provide translational context for the selected doses, the human equivalent dose (HED) was additionally estimated using body surface area normalization (Reagan‐Shaw et al. 2008). The highest dose used in this study (200 mg/kg/day in mice) corresponds to an HED of approximately 16.2 mg/kg/day, equivalent to approximately 0.973 g/day for a 60‐kg adult. In addition, PS was used as a positive control, considering previous evidence demonstrating its anti‐photoaging effects in UV‐induced skin damage, which has been reported to prevent the UV‐induced reduction of type I procollagen and inhibit UV‐induced MMP‐1 expression in human dermal fibroblasts and in vivo human skin (Cho et al. 2008). Moreover, oral supplementation with PS reduced wrinkle formation and skin thickness and improved skin moisture and collagen content in UV‐irradiated hairless mice (Choi et al. 2013). Therefore, PS was considered an appropriate reference compound for evaluating anti‐photoaging efficacy in our study.

### 2.5 Skin Thickness, Wrinkle, and Moisture Ratio Analysis

To evaluate skin thickness, a double layer of skin thickness was measured using a digital caliper (CD‐15APX, Mitutoyo, Japan) at the end of the experiment. Skin thickness was evaluated by holding a 1‐cm‐wide section below the center of the HR‐1 mouse back skin. To evaluate skin moisture, the moisture ratio was estimated on the back of HR‐1 mice using a skin moisture content analysis device (WillCam, K.L Global, Seoul, Korea). Wrinkle formation on the dorsal skin of the mice was assessed using a skin replica kit (Epigem Inc., Seoul, Korea). Skin replicates were obtained and analyzed to measure wrinkles using a device (Visioline VL650, CK Electronics GmbH, Cologne, Germany). The assessment of skin wrinkles included the total wrinkle length, wrinkle area, and mean wrinkle depth (Park et al. 2017).

### 2.6 Histological Analysis

Dorsal skin tissues were obtained from HR‐1 mice and fixed in 10% formalin for histological analysis. After embedding the skin tissues in paraffin, the paraffin blocks were sliced and stained with hematoxylin and eosin (H&E) for epidermal thickness analysis or with Masson's trichrome staining for collagen fiber analysis (Liu et al. 2024). Immunohistochemistry (IHC) was conducted using an AGEs antibody to analyze the accumulation of AGEs in skin tissue. Images of stained sections were obtained, and epidermal thickness was analyzed using a digital slide scanner (Motic Easyscan One, Motic, Hong Kong) and image analysis software (Motic DS Assistant, Hong Kong).

### 2.7 Enzyme‐Linked Immunosorbent Assay (ELISA) Analysis

The levels of aspartate aminotransferase (AST) and alanine aminotransferase (ALT) in the serum and tissue inhibitor of metalloproteinases 1 (TIMP1), HYAL1, HYAL2, and hyaluronan in the dorsal skin of HR‐1 mice were detected using ELISA. The levels of AST and ALT in the serum of HR‐1 mice were measured using commercial ELISA kits, an AST activity assay kit (MAK055; Sigma‐Aldrich, MO, USA), and an ALT activity assay kit (MAK052; Sigma‐Aldrich, USA). The expression of TIMP1, HYAL1, HYAL2, and hyaluronan in the skin tissue was measured using a TIMP1 ELISA kit (ab196265; Abcam, CA, UK), hyaluronidase‐1 ELISA kit (MBS9395469; MyBiosource, CA, USA), hyaluronidase‐2 ELISA kit (MBS28865; MyBiosource, CA, USA), and hyaluronan Quantikine ELISA Kit (DHYAL0; R&D Systems, MN, USA) according to the manufacturer's instructions.

### 2.8 Western Blotting

Skin tissues were lysed using RIPA buffer (Cell Signaling Technology, Danvers, MA, USA) containing phosphatase and protease inhibitors (Sigma‐Aldrich, MO, USA). The lysed tissues were quantified using a DC Protein Assay Kit (Bio‐Rad, Hercules, CA, USA). Equal amounts of protein were separated using 8%–12% sodium dodecyl sulfate‐polyacrylamide gel electrophoresis. Separated proteins were electrotransferred onto an immunoblotting nitrocellulose membrane (Bio‐Rad). The membranes were blocked with 5% skim milk in Tris‐buffered saline containing 0.2% Tween‐20 (TBST) at room temperature for 1 h. After the membranes were blocked, they were incubated with each primary antibody for proteins at 4°C for 18 h. After incubation with antibodies, the membranes were washed with TBST for 5 min, repeated three times. Each membrane was then incubated with appropriate horseradish peroxidase‐conjugated secondary antibodies at room temperature for 1 h. After incubation, the blots were visualized using an enhanced chemiluminescence detection reagent (Lumi Femto Solution; Dogen Bio, Seoul, Korea) and imaged using a ChemiDoc XRS+ imaging system (Bio‐Rad). The density of each band was analyzed using Image Lab software (Version 4.1, Bio‐Rad), and β‐actin was used as a housekeeping control.

### 2.9 Statistical Analysis

All results are presented as mean ± standard error of the mean (SEM). Statistical analyses were performed using GraphPad Prism 9.0 software (La Jolla, CA, USA). One‐way analysis of variance (ANOVA) followed by Dunnett's post hoc test was used for multiple comparisons. A *p* value < 0.05 was considered statistically significant.

## 3 Results

### 3.1 HPLC Analysis of RR‐r Extract

The concentration of the reference compound in RR‐r extract was 2.2166%. Accordingly, the proposed HPLC method was validated by determining its linearity, limit of detection and quantification, inter‐ and intraday precision, repeatability, stability, and recovery (Table 1). These results demonstrate the suitability of the developed HPLC method for analyzing the constituents of RR‐r extract. As shown in Figure 1, the peaks represent the chromatogram of the reference compound and the chemical composition of the RR‐r extract. The calibration curve was generated using standard rutin at concentrations between 0.125 and 0.5 (mean ± SD) μg/mL.

**TABLE 1:** Validation and evaluation of high‐performance liquid chromatography.

Analyte	Regression equation	Correlation coefficient	Linear range (μg/μL)	LOD^a^ (μg)	LOQ^b^ (μg)
Rutin	y = 4,803, 828.3570 x + 244,950.6250	*R* ^2^ = 0.9999	0.03125–0.50000	0.00563	0.01707

Analyte	Precision RSD^c^				
	Concentration (μg/mL)	Intra‐day (*n* = 3)	Inter‐day (*n* = 3)	Repeat‐ability RSD (%, *n* = 5)	
Rutin	0.5	0.019	0.124	2.416	
	0.25	0.1	0.183		
	0.125	0.252	0.106		

Sam:Std	Accuracy				
	Spiked (μg)	Detected (μg)	Recovery (%)	RSD (%)	
1:02	2.406	2.367	98.387	2.079	
1:01	2.358	2.355	99.865	0.039	
2:01	2.311	2.332	100.901	2.054	

^a^ Limit of detection.

^b^ Limit of quantification.

^c^ Relative standard deviation.

**FIGURE 1:**
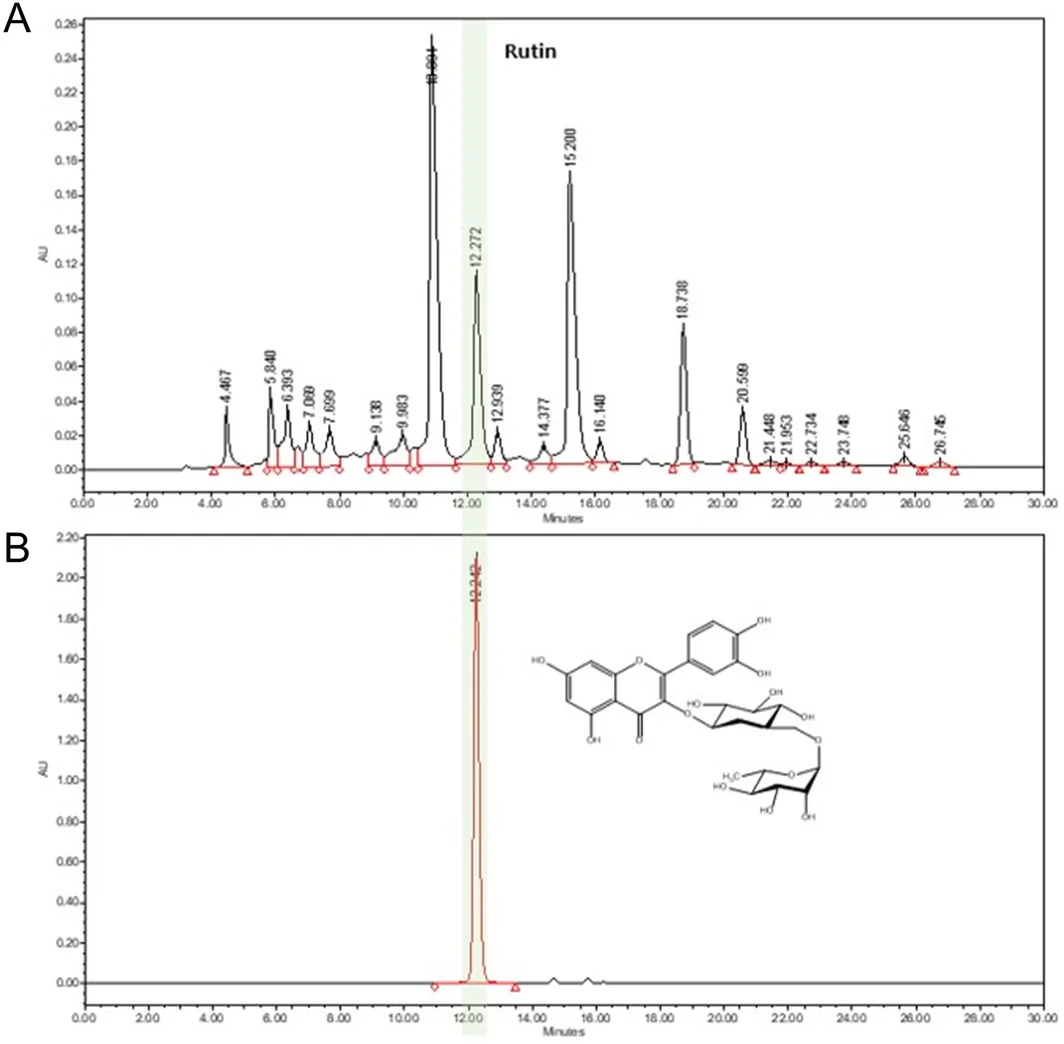
HPLC chromatographic profile of RR‐r extract. Representative high‐performance liquid chromatography–ultraviolet (HPLC–UV) chromatograms of (A) RR‐r extract and (B) the rutin reference standard, recorded at 254 nm.

### 3.2 Change of Body Weight and Liver Function by RR‐r Extract

Body weight and serum AST and ALT levels were measured at the end of the experiment to investigate the effects of RR‐r on body weight and serum liver function. As shown in Figure 2A, the body weights of mice did not significantly differ among the groups. In addition, the serum levels of ALT and AST were not significantly different between the groups (Figure 2B,C). Therefore, the results suggest that the RR‐r extract did not affect by changes in body weight or serum levels of AST and ALT in HR‐1 mice.

**FIGURE 2:**
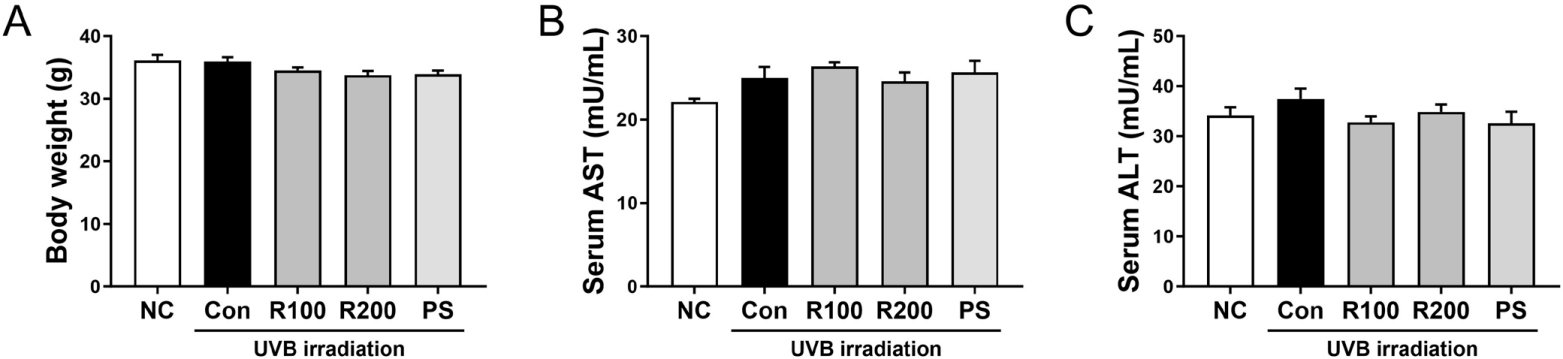
Effect of RR‐r extract on body weight and serum markers of hepatic toxicity in UVB‐irradiated hairless mice. (A) Final body weight at the end of the experimental period. Serum levels of (B) aspartate aminotransferase (AST) and (C) alanine aminotransferase (ALT) levels. Data are presented as the mean ± SEM. (*n* = 8).

### 3.3 Alleviation Effect of RR‐r on Increasing the Skin Wrinkle Formation and Thickness by UVB Irradiation

Skin replica and H&E staining were performed on the back skin of HR‐1 mice to observe the effect of RR‐r on wrinkle formation and changes in skin thickness. The UVB‐irradiated control group showed remarkable wrinkles compared with the NC group (Figure 3A). However, there was a tendency for reduced wrinkle formation in the RR‐r orally administered group compared to that in the UVB control group. In addition, the total wrinkle length, wrinkle area, and mean wrinkle depth showed decreasing trends in the treatment group, and the total wrinkle length (36%) and wrinkle area (44.6%) were significantly reduced in the R200 group (*p* = 0.037 and *p* = 0.010; Figure 3B,C) respectively compared to the UVB‐irradiated control group; however, mean wrinkle depth did not show a significant difference (Figure 3D). Furthermore, as shown in Figure 3E, epidermal skin thickness was less extended in the R100 and R200 groups compared with the UVB control group in the H&E staining results, and was significantly decreased in the R200 group (30.8%) compared to the UVB control group (*p* = 0.001; Figure 3F). Basal skin thickness was significantly reduced by 23.1% in the R200 group compared to that in the UVB‐irradiated control group (*p* = 0.004; Figure 3G). Consequently, these results indicate that RR‐r extract prevents increased wrinkle formation and skin thickness induced by UVB irradiation in the skin.

**FIGURE 3:**
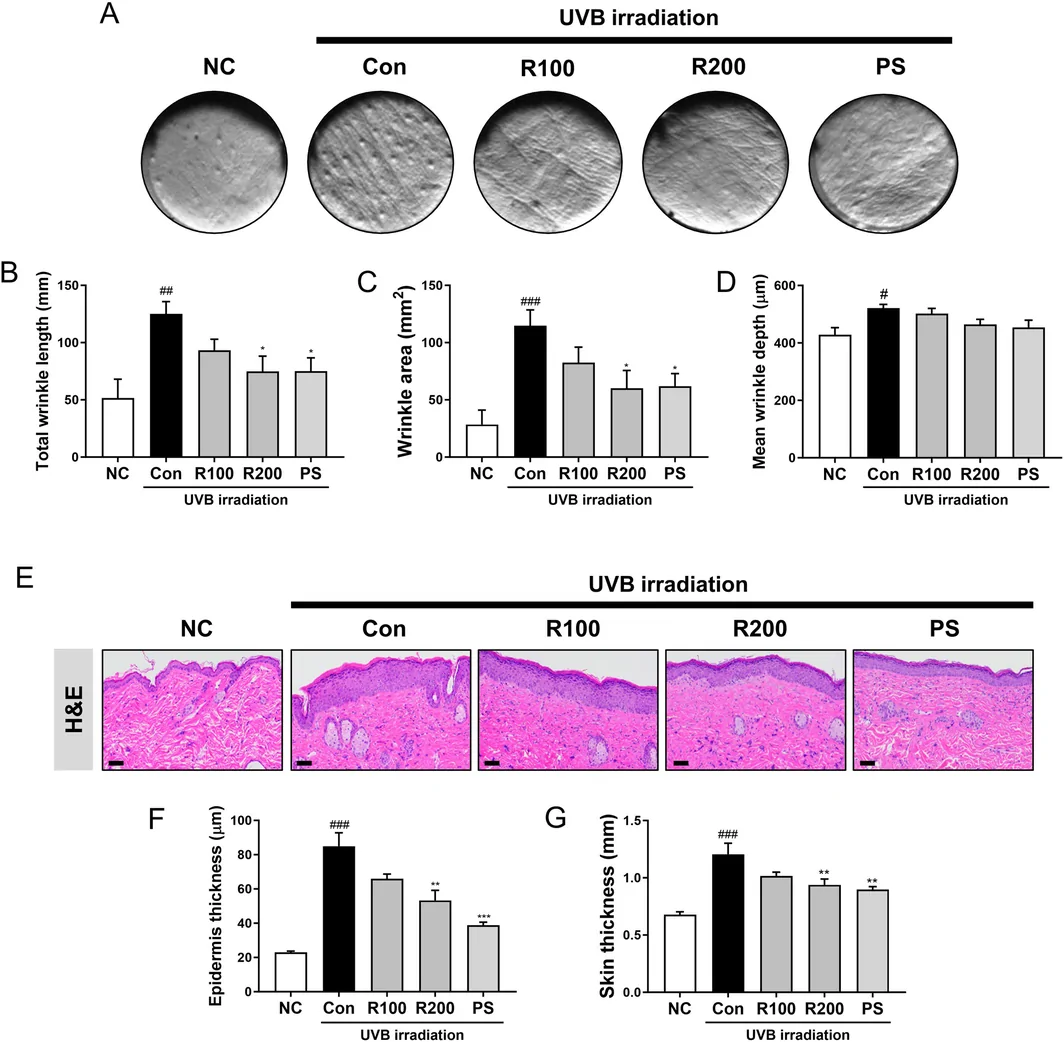
Effects of RR‐r extract on UVB‐induced wrinkle formation and skin thickening in hairless mice. (A) Representative images of dorsal skin replicas. Quantification of (B) total wrinkle length, (C) wrinkle area, and (D) mean wrinkle depth. (E) Representative images of hematoxylin and eosin (H&E)‐stained dorsal skin sections. Scale bar = 50 μm. Quantification of (F) epidermal thickness and (G) dorsal skin thickness. For the histological analysis, three randomly selected, non‐overlapping fields were evaluated for each mouse, and the mean value was used as the representative value for that mouse. Data are presented as the mean ± SEM (*n* = 8 mice per group). ^#^ *p* < 0.05, ^##^ *p* < 0.01, and ^###^ *p* < 0.001 versus the normal control group; *p < 0.05, **p < 0.01, and ***p < 0.001 versus the UVB‐irradiated control group.

### 3.4 Regulation of Collagen and Skin Wrinkle‐related Proteins by RR‐r Extract Under UVB‐Irradiation

In the control group, collagen fiber areas stained with Masson's trichrome were reduced in compared with the NC group; however, the degradation of collagen fibers was prevented by the oral administration of RR‐r extract (Figure 4A). In addition, the expression of pro‐collagen type I A1, a target of MMP1, was lower in the UVB control group than in the NC group; however, the level was significantly higher by 2.25‐fold in the R200 group than in the UVB control group (*p* = 0.003; Figure 4B). TIMP1, an inhibitor of MMPs, was increased 1.57‐fold in both the R100 and R200 group compared with the UVB control group (*p* = 0.005 and *p* = 0.005; Figure 4C). Furthermore, the effect of RR‐r on the expression of MMP1, MMP3, and MMP9 in the skin tissues of HR‐1 mice after UVB irradiation was confirmed by Western blotting. MMP1, MMP3, and MMP9 protein expression levels were decreased after RR‐r administration, and significant differences were observed in the R200 group, with decreases of 37.7%, 51.1%, and 66.1% compared with the UVB control group, respectively (*p* = 0.0313, *p* < 0.001, and *p* < 0.001; Figure 4D). These results suggest that RR‐r administration alleviates wrinkle formation in the skin caused by UVB‐induced photoaging.

**FIGURE 4:**
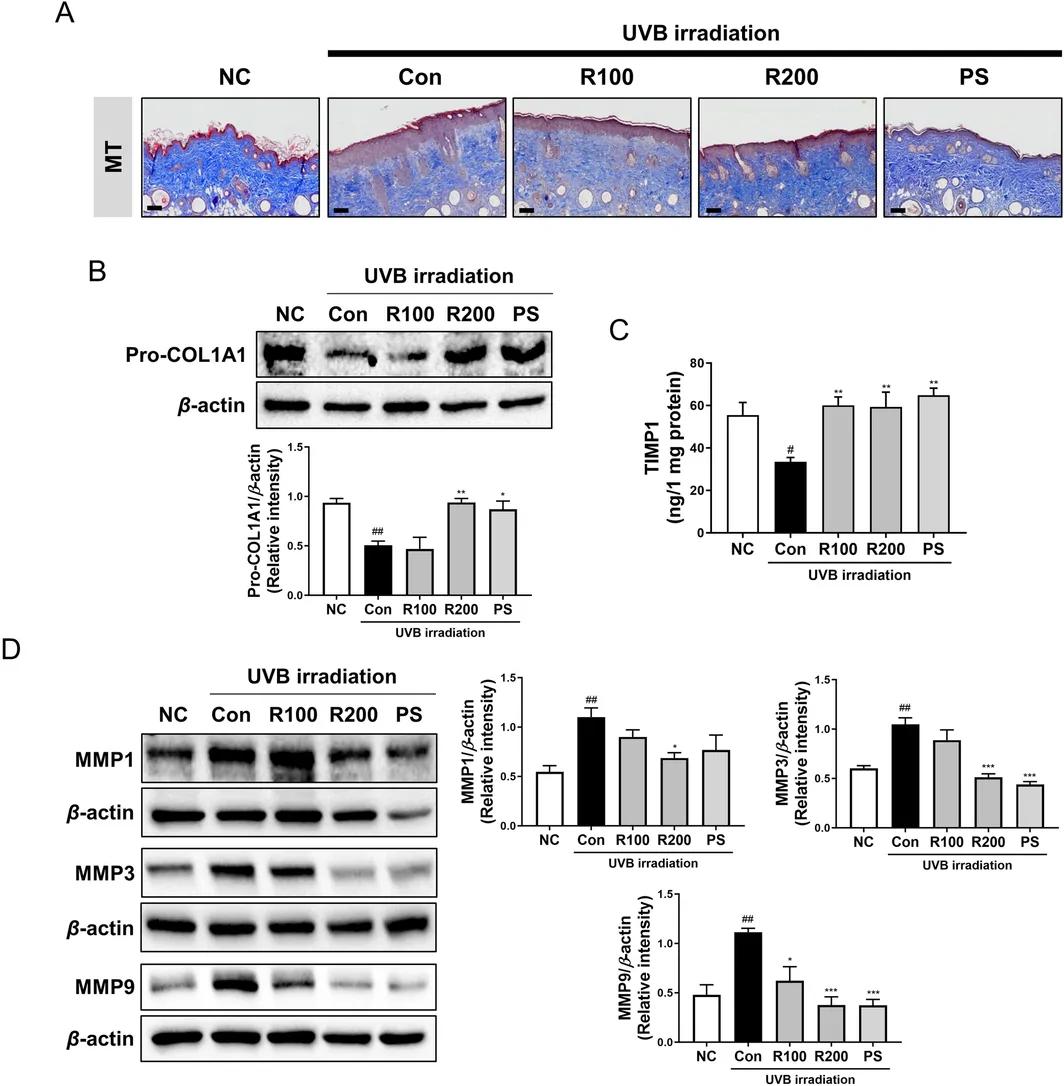
Effects of RR‐r extract on UVB‐induced collagen degradation and extracellular matrix remodeling in the skin of hairless mice. (A) Representative images of Masson's trichrome‐stained dorsal skin sections showing collagen fibers. Scale bar = 50 μm. Three randomly selected, non‐overlapping fields were evaluated for each mouse. (B) Protein expression of pro‐collagen type I A1 in skin tissue with densitometric quantification. (C) Tissue inhibitor of metalloproteinase 1 (TIMP‐1) levels in skin tissue. (D) Protein expression of matrix metalloproteinases (MMP1, MMP3, and MMP9) in skin tissue with densitometric quantification. Data are presented as the mean ± SEM (*n* = 3 mice per group). ^#^ *p* < 0.05, ^##^ *p* < 0.01, and ^###^ *p* < 0.001 versus the normal control group; *p < 0.05, **p < 0.01, and ***p < 0.001 versus the UVB‐irradiated control group.

### 3.5 Changes of Skin Hydration‐regulated Proteins by RR‐r Administration Under UVB‐Irradiation

The skin moisture ratio was measured in the dorsal skin of HR‐1 mice. UVB irradiation decreased the moisture ratio; however, the R100 and R200 groups showed a significantly increased skin moisture ratio by 21.1% and 21.2% in compared with the UVB control group, respectively (*p* = 0.010 and *p* = 0.009; Figure 5A). Next, we confirmed whether RR‐r prevented UVB‐induced skin dehydration using ELISA and Western blotting. Quantification of hyaluronan in the skin tissue of HR‐1 mice treated with RR‐r extract (100 mg/kg and 200 mg/kg) showed significantly increased levels in a dose‐dependent manner (1.44‐ and 2.08‐fold) following RR‐r administration compared with those in the UVB control group (*p* = 0.019 and *p* < 0.001; Figure 5B). In addition, the levels of HYAL1 and HYAL2 in the skin tissue of HR‐1 mice decreased in the RR‐r administration groups compared with the UVB control group; in particular, the R200 group showed significant differences of 41% and 16.7% (*p* = 0.012 and *p* = 0.002; Figure 5C,D). Furthermore, in the R200 group, the expression of HAS enzymes (1, 2, and 3), filaggrin, skin hydration‐regulated proteins, and collagen‐1 was consistent with the hyaluronan results, with increases of 2.4‐, 4.1‐, 1.5‐, 16.4‐, and 2.5‐fold compared with the UVB control group, respectively (*p* = 0.0379, *p* = 0.009, *p* < 0.001, and *p* = 0.0018; Figure 5E). Taken together, these results suggest that RR‐r effectively regulates skin hydration‐regulated proteins by stimulating the HAS enzymes, filaggrin, and collagen‐1, and the synthesis of hyaluronan, while suppressing HYALs in the skin under UVB irradiation.

**FIGURE 5:**
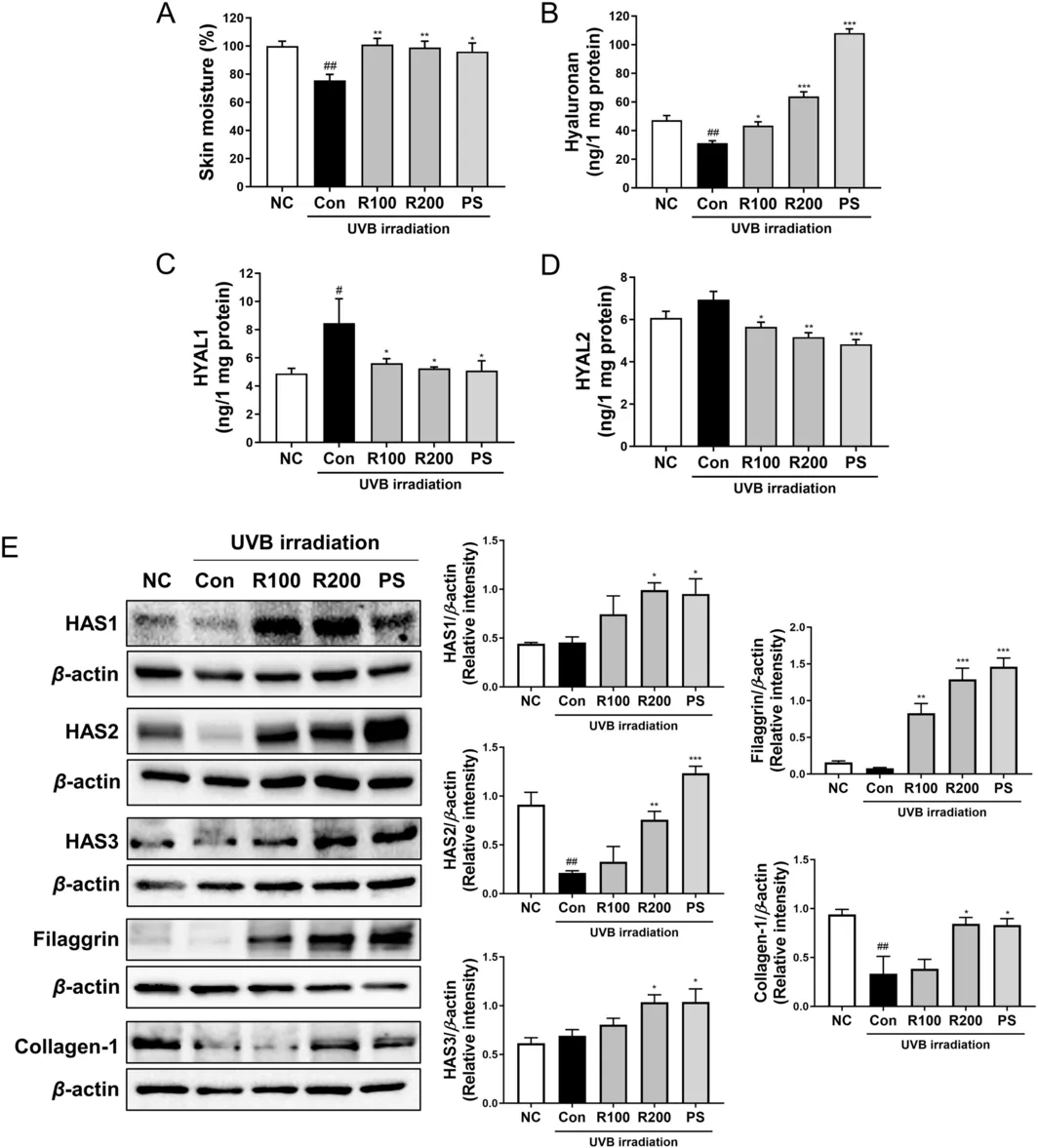
Effects of RR‐r extract on UVB‐induced alterations in skin hydration and hydration‐related proteins in hairless mice. (A) Dorsal skin moisture content. Skin tissue levels of (B) hyaluronan, (C) hyaluronidase 1 (HYAL1), and (D) hyaluronidase 2 (HYAL2). (E) Protein expression of hyaluronan synthases (HAS1, HAS2, and HAS3), filaggrin, and collagen type I A1 in skin tissue with densitometric quantification. Data are presented as the mean ± SEM (*n* = 8 mice per group for A–D and *n* = 3 mice per group for E). ^#^ *p* < 0.05, ^##^ *p* < 0.01, and ^###^ *p* < 0.001 versus the normal control group; *p < 0.05, **p < 0.01, and ***p < 0.001 versus the UVB‐irradiated control group.

### 3.6 Reduction of MAPKs and AP‐1 Expression by RR‐r Administration Under UVB Irradiation

UV irradiation causes the activation of AP‐1, which is closely dependent on the activation of the MAPK signaling pathway in skin aging (Laure and Gary 2002). The UVB control group showed significantly increased phosphorylation levels of MAPKs, including p38, JNK, and ERK, compared to the NC group (*p* < 0.001, *p =* 0.011, and *p =* 0.007; Figure 6A). In contrast, the R200 group showed decreased phosphorylation levels of p38 and ERK by 52.8% and 66.2% compared to the UVB control group (*p* < 0.001 and *p* = 0.0276); however, p‐JNK levels did not show a significant difference (*p* = 0.0146). As shown in Figure 6B, the UVB control group exhibited higher c‐Fos and c‐Jun phosphorylation levels than the normal control group; however, a significant difference was observed in c‐Fos phosphorylation (*p* = 0.001). In contrast, the RR‐r 200 mg/kg group demonstrated decreased c‐Fos and c‐Jun phosphorylation by 40.3% and 34.8% compared with the UVB control group (*p* = 0.01 and *p* = 0.016). These findings indicates that the administration of RR‐r extract may play a role in the prevention of UVB‐induced skin damage by decreasing the activation of MAPKs and the c‐Fos/c‐Jun signaling pathway.

**FIGURE 6:**
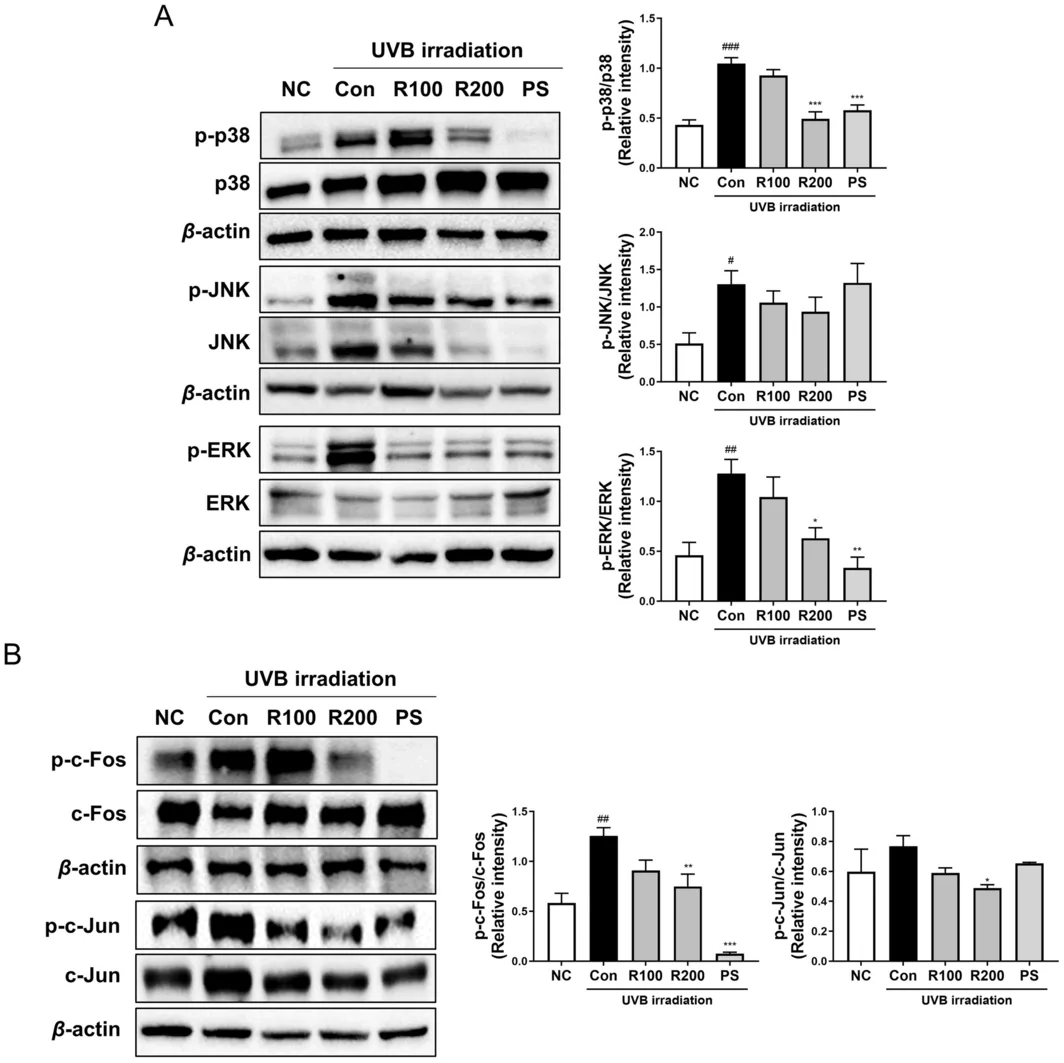
Effects of RR‐r extract on UVB‐induced activation of the MAPK/AP‐1 signaling pathway in the skin of hairless mice. (A) Protein expression and phosphorylation of p38 mitogen‐activated protein kinase (p38), c‐Jun N‐terminal kinase (JNK), and extracellular signal‐regulated kinase (ERK) in skin tissue with densitometric quantification. Phosphorylated protein levels were normalized to the corresponding total protein levels. (B) Protein expression and phosphorylation of the activator protein‐1 components c‐Fos and c‐Jun in skin tissue with densitometric quantification. Phosphorylated protein levels were normalized to the corresponding total protein levels. Data are presented as the mean ± SEM (*n* = 3 mice per group). ^#^ *p* < 0.05, ^##^ *p* < 0.01, and ^###^ *p* < 0.001 versus the normal control group; *p < 0.05, **p < 0.01, and ***p < 0.001 versus the UVB‐irradiated control group.

### 3.7 Changes of AGE‐RAGE Expression by RR‐r Administration Under UVB Irradiation

At the molecular level, there is growing evidence that AGEs are one of the markers of skin photoaging (Geng et al. 2021). In this study, we conducted IHC analysis and Western blotting to evaluate the expression of AGEs and RAGE in the skin tissues of HR‐1 mice. As shown in Figure 7A, we observed an increase in AGE intensity in the UVB‐irradiated control group compared with the NC group; however, the RR‐r‐treated groups (100 mg/kg and 200 mg/kg) showed lower AGE intensity in the skin tissue compared with the UVB‐irradiated control group. In addition, as shown in Figure 7B, the expression levels of AGE and RAGE in the skin tissue were significantly increased by 1.71‐ and 1.85‐fold in the UVB‐irradiated control group compared with those in the NC group (*p* = 0.034 and *p* = 0.016). These result were consistent with the IHC analysis, in which the level of expression was significantly reduced following 100 mg/kg and 200 mg/kg RR‐r administration under UVB irradiation by 39.4% and 54.6% compared with that in the UVB group (*p* = 0.043 and *p* = 0.008), respectively. The levels of RAGE expression showed significant differences in the R100 and R200 groups, with decreases of 37.6% and 57.3% compared with the UVB control group (*p* = 0.041 and *p* = 0.005). Therefore, these results suggest that the administration of RR‐r suppressed the expression of AGEs and RAGE associated with UVB‐induced AGE‐RAGE axis activation in skin tissue.

**FIGURE 7:**
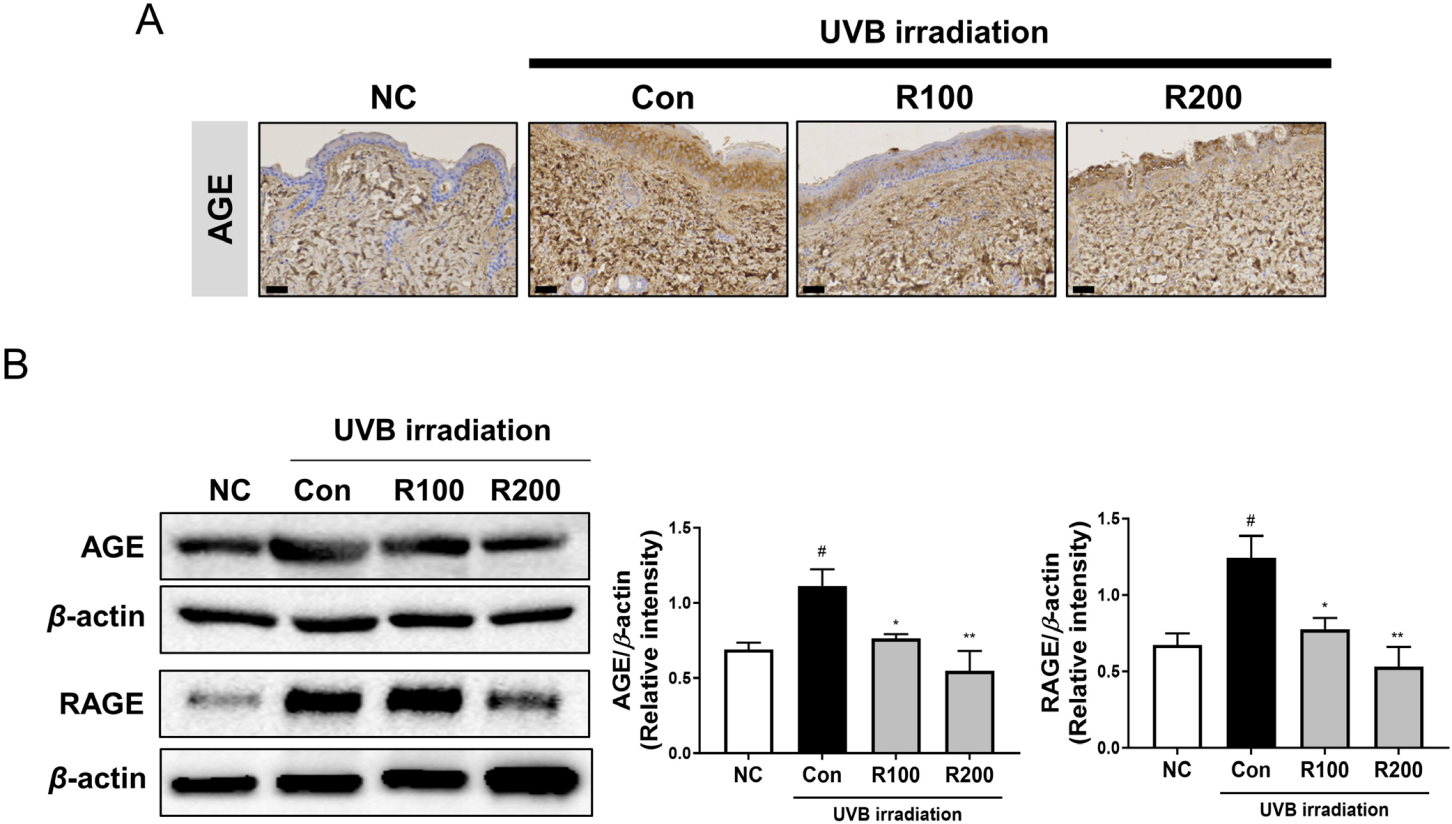
Effects of RR‐r extract on UVB‐induced accumulation of advanced glycation end‐products and RAGE expression in the skin of hairless mice. (A) Representative immunohistochemical images showing the accumulation of advanced glycation end‐products (AGEs) in dorsal skin tissue. Scale bar = 50 μm. Three randomly selected, non‐overlapping fields were evaluated for each mouse. (B) Protein expression of AGEs and the receptor for advanced glycation end products (RAGE) in skin tissue with densitometric quantification. Data are presented as the mean ± SEM (*n* = 3 mice per group). ^#^ *p* < 0.05 versus the normal control group; *p < 0.05 and **p < 0.01 versus the UVB‐irradiated control group.

## 4 Discussion

RR‐r is a traditional medicinal plant widely used as an herbal medicine for various diseases (An et al. 2011; Choi et al. 2022; Park et al. 2025). Although the flowers and fruits of *Rosa rugosa* have been extensively studied and used in traditional remedies, recent pharmacological investigations have increasingly focused on the bioactive potential of its roots, uncovering a range of biological activities (Zhang et al. 2024). RR‐r has anti‐inflammatory, antioxidant, and hepatoprotective effects (Cheol Park et al. 2004). However, the anti‐photoaging and dermatological benefits of RR‐r remain unclear. This study aimed to investigate the protective effects of RR‐r against UVB‐induced photoaging and the associated molecular changes in HR‐1 hairless mice.

First, the quality and consistency of the RR‐r extract were confirmed by HPLC analysis, which identified rutin as the major reference compound, present at a concentration of 2.2166%. The developed analytical method was rigorously validated for linearity, precision, detection limits, stability, and recovery, thereby ensuring its reliability for quantifying bioactive compounds in RR‐r. This is a critical step in standardizing the herbal extracts and supporting their reproducibility in future pharmacological studies. Administration of RR‐r significantly ameliorated various UVB‐induced alterations, including skin wrinkle formation, epidermal thickening, collagen degradation, skin dehydration, oxidative stress, and the activation of pro‐aging signaling pathways. Importantly, RR‐r did not induce significant changes in body weight or AST and ALT levels, suggesting that RR‐r was well tolerated when administered orally up to 200 mg/kg. These results support the safety of root extracts.

One of the hallmark features of skin photoaging is the formation of deep wrinkles and increased epidermal thickness due to prolonged UV exposure (Gao et al. 2025). In our study, RR‐r treatment led to a marked reduction in total wrinkle length, wrinkle area, and mean wrinkle depth compared with the UVB‐irradiated control group. Histological analysis confirmed that RR‐r attenuated UVB‐induced epidermal hyperplasia and basal skin thickening. These results indicate that RR‐r exhibits anti‐wrinkle and skin‐preserving properties. Collagen degradation by MMPs is another critical process that underlies skin photoaging (Hu et al. 2025). UVB exposure upregulated the expression of MMP1, MMP3, and MMP9 in the mouse skin, consistent with their known roles in the degradation of ECM components, particularly collagen. Notably, RR‐r administration significantly reduced the expression of MMPs and concurrently increased the levels of TIMP‐1, an endogenous inhibitor of MMPs. Additionally, RR‐r restored pro‐collagen type I A1 levels, indicating the recovery of collagen biosynthesis. Collectively, these results suggest that RR‐r modulates the balance between matrix degradation and regeneration, thereby preserving dermal integrity.

Skin hydration is another essential aspect of healthy skin, often compromised by UVB‐induced damage (Xiang et al. 2024). The present study demonstrated that RR‐r not only increased skin moisture content, but also elevated the expression of key hydration‐related proteins, including HAS enzymes, filaggrin, and collagen‐1. Moreover, RR‐r significantly decreased the expression of hyaluronidases (HYAL1 and HYAL2), which degrade hyaluronan. These findings suggest that RR‐r promotes water retention in the skin by enhancing hyaluronan synthesis and reducing its degradation, thus improving the overall skin barrier function and resilience.

The MAPK pathways, including p38, JNK, and ERK, are major mediators of UVB‐induced skin damage (J. H. Kim et al. 2025). Their activation leads to the stimulation of AP‐1 transcription factors, such as c‐Fos and c‐Jun, which subsequently upregulate MMPs and inflammatory cytokines (J. H. Kim et al. 2025). In our study, RR‐r significantly suppressed the phosphorylation of MAPKs and downstream AP‐1 components. This suggests that RR‐r exerts its anti‐photoaging effects by attenuating UVB‐induced stress signaling pathways and preventing the overexpression of pro‐aging genes and inflammation‐related proteins. Furthermore, AGEs accumulate in the skin owing to chronic UV exposure and interact with RAGE, leading to oxidative stress, inflammation, and cellular senescence (Gu et al. 2023). Therefore, we examined the AGE‐RAGE axis, which has gained attention as a novel mechanism contributing to skin aging. Our findings revealed that RR‐r markedly suppressed AGEs accumulation and RAGE expression in UVB‐irradiated skin tissue, as confirmed by immunohistochemistry and Western blotting. This inhibitory effect on the AGE‐RAGE pathway provides further evidence for the antioxidative and anti‐inflammatory potential of RR‐r in the context of skin photoaging. However, the specific active constituents responsible for these effects have not yet been fully characterized. Clinical trials are necessary to confirm the efficacy and safety of RR‐r in human subjects with photoaged skin. The present results suggest that RR‐r mediates its protective effects against UVB‐induced skin damage through a multi‐target approach. It mitigates morphological changes, such as wrinkle formation and epidermal thickening, restores collagen homeostasis, enhances skin hydration, and modulates key intracellular signaling pathways, including the MAPK/AP‐1 and AGE‐RAGE axes. These comprehensive actions are likely mediated by the synergistic activity of various bioactive compounds within the extract, including flavonoids, such as rutin.

Despite these promising findings, this study had several limitations. First, it relied on a single UVB‐induced photoaging model using HR‐1 hairless mice, which may not fully recapitulate the complexity of human skin aging. Although this model is widely used to investigate photoaging mechanisms, species‐specific differences in skin physiology may limit its direct translation to humans. Second, this study did not include human clinical or ex vivo skin experiments, which are necessary to confirm the relevance of RR‐r in human skin. Third, although we demonstrated the modulation of the MAPK/AP‐1 and AGE‐RAGE signaling pathways, additional mechanistic studies are required to establish direct molecular targets. Future investigations, including human trials and mechanistic validation studies, are necessary to further support the translational potential of RR‐r.

## 5 Conclusions

RR‐r attenuates UVB‐induced skin photoaging in HR‐1 hairless mice by reducing wrinkle formation, restoring collagen homeostasis, and enhancing skin hydration. These protective effects were associated with modulation of the MAPK/AP‐1 and AGE‐RAGE signaling pathways, suggesting a multi‐targeted mechanism of action. Although the specific active constituents remain to be identified and clinical validation is required, the present findings provide experimental evidence supporting the potential of RR‐r as a natural candidate for mitigating UVB‐induced skin aging. Future studies should focus on characterizing the active compounds and confirming their efficacy and safety in human models before their practical application as functional foods or cosmeceutical ingredients.

## Author Contributions

**Min Ji Gu:** writing – original draft, writing – review and editing, formal analysis. **Guijae Yoo:** writing – original draft, formal analysis. **Sang‐Hoon Lee:** funding acquisition, investigation, resources. **Ho‐Young Park:** investigation, resources. **Yoonsook Kim:** funding acquisition, investigation, resources. **Donghwan Kim:** writing – original draft, writing – review and editing, investigation. **Sang Keun Ha:** writing – review and editing, conceptualization, funding acquisition, project administration.

## Funding

This research was supported by Main Research Program (E0210203) of the Korea Food Research Institute (KFRI) funded by the Ministry of Science and ICT (MSIT).

## Ethics Statement

An experiment was conducted under guidelines of the ethical committee for Animal Care and Use according to an animal protocol (approval no. IV‐RA‐16‐2006‐22), and complied with the Laboratory Animals Welfare Act and the Korean national laws for animal welfare.

## Conflicts of Interest

The authors declare no conflicts of interest.

## Data Availability

The data that support the findings of this study are available from the corresponding author upon reasonable request.
